# Lateral Tension-Induced Penetration of Particles into a Liposome

**DOI:** 10.3390/ma10070765

**Published:** 2017-07-07

**Authors:** Kazuki Shigyou, Ken H. Nagai, Tsutomu Hamada

**Affiliations:** 1 School of Materials Science, Japan Advanced Institute of Science and Technology, 1-1 Asahidai, Nomi, Ishikawa 923-1292, Japan; s1440007@jaist.ac.jp; 2 Bio-AFM Frontier Research Center, Institute of Science and Engineering, Kanazawa University, Kakuma-machi, Kanazawa 920-1192, Japan

**Keywords:** particle penetration, lateral tension, lipid membrane, gene delivery method

## Abstract

It is important that we understand the mechanism of the penetration of particles into a living cell to achieve advances in bionanotechnology, such as for treatment, visualization within a cell, and genetic modification. Although there have been many studies on the application of functional particles to cells, the basic mechanism of penetration across a biological membrane is still poorly understood. Here we used a model membrane system to demonstrate that lateral membrane tension drives particle penetration across a lipid bilayer. After the application of osmotic pressure, fully wrapped particles on a liposome surface were found to enter the liposome. We discuss the mechanism of the tension-induced penetration in terms of narrow constriction of the membrane at the neck part. The present findings are expected to provide insight into the application of particles to biological systems.

## 1. Introduction

Nano/submicron particles have been actively studied because of their potential for controlling living cells, such as for drug delivery [1,2,3,4,5], local heating [6], and the visualization of proteins [7]. To express these functions, particles need to be transported into cells, and this process normally involves cellular uptake functions that are controlled by proteins [8]. Cells wrap particles within their membranes, and membrane-wrapped particles then enter the cells through fission of the membrane.

Membrane fission in a living cell has been shown to be controlled by dynamin proteins, which induce bilayer constriction and lateral tension to achieve fission of the tube (see Figure 5 for a definition of “neck” and “tube”) [9,10,11,12]. These results imply that the application of lateral tension at a constricted bilayer can promote the penetration of particles through a membrane. We previously reported a high-efficiency particle penetration system that did not involve proteins, where the self-penetration of oxide carbon particles on artificial lipid vesicles was observed [13]. The particles tended to be wrapped by membranes when the particles strongly adhered to the membrane surface. Excess surface area of the membrane was then decreased to create wrapping regions. Due to the increase in lateral tension caused by the wrapping of numerous particles, the membrane could be cut at the neck region of fully wrapped particles.

In this study, we demonstrate particle penetration through a membrane induced by an increase in membrane lateral tension using a cell-sized liposome without the aid of proteins. Previously, we reported the preparation of liposomes with fully wrapped or partially wrapped particles with the use of centrifugal force [14]. In this experiment, we used fully wrapped particles. Lateral tension drives the release of wrapped particles from the mother liposome.

## 2. Results

**Figure 1:**
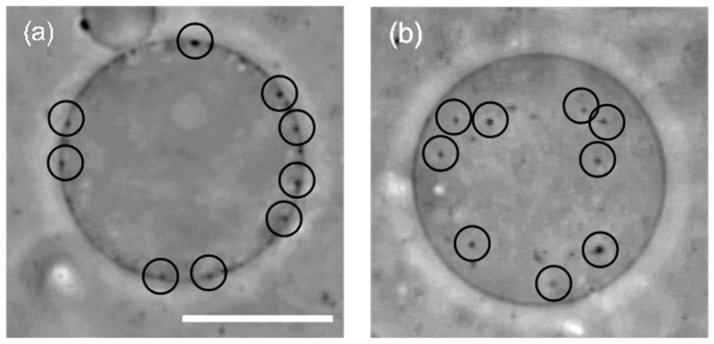
(**a**) A liposome with 200 nm fully wrapped particles before the application of osmotic pressure. Black circles show the position of fully wrapped particles; (**b**) Penetration of particles after the application of osmotic pressure. Scale bar is 10 µm.

**Figure 2:**
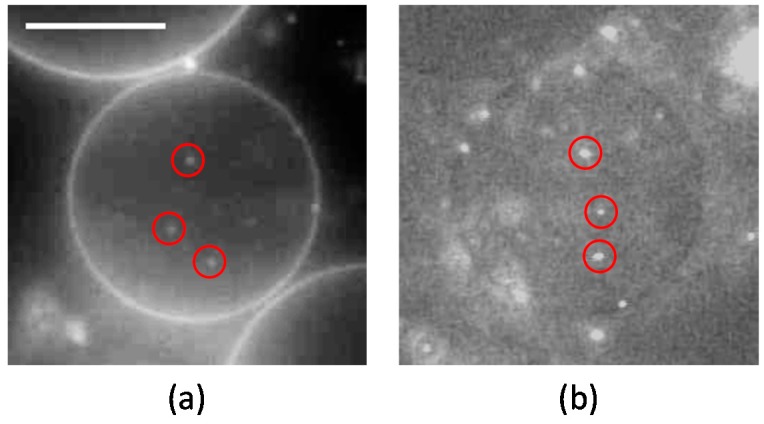
Microscopic images of (**a**) fluorescent lipid rho-DHPE (N-(rhodamine red-X)-1,2-dihexadecanoyl-sn-glycero-3-phosphoethanolamine triethylammonium salt) and (**b**) YG-particle fluorescence. The red circles indicate penetrated particles. Scale bar is 10 μm.

First, we prepared liposomes with fully wrapped particles by applying centrifugal force to the mixture of liposomes and particles (the detailed procedure is indicated in the Materials and Methods section) [14]. Next, we observed the response of the particles to the application of lateral tension under a decrease of the sucrose concentration in the bulk solution (a video of this penetration is available in the SI). Figure 1a shows a phase-contrast image of wrapped particles on the liposome before the application of lateral tension. Each particle is indicated by a circle. After the application of lateral tension, the particles penetrated into the liposome (Figure 1b). In addition, an experiment with a fluorescent lipid shows that penetrated particles were covered by the membrane (Figure 2). This implies that the penetration was caused by membrane scission at the neck of a fully wrapped particle.

**Figure 3:**
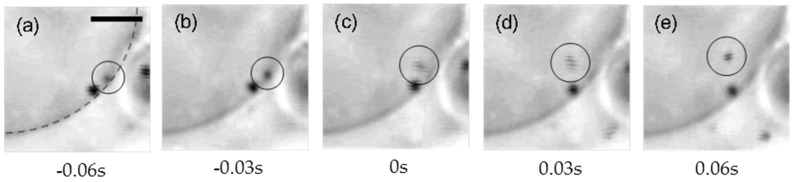
(**a**–**e**) Time-lapse images of the particle-uptake into a liposome (the time 0 s means the initiation of the particle penetration.). The black circle surrounds a 200 nm fully wrapped particle. The gray broken line shows the edge of the liposome. Scale bar is 10 µm.

Next, we focused on the equatorial plane of the liposomes to observe the dynamic process of particle penetration. Figure 3 shows time-lapse images of the penetration of a wrapped particle under the application of lateral tension (calculation of the lateral tension is mentioned in the Materials and Methods section). Membrane fission occurred within 0.03 s. Notably, we did not observe any structural connection between the mother liposome and the penetrated particles after fission.

**Figure 4:**
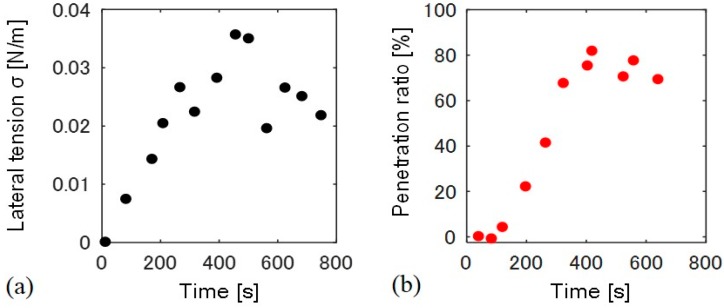
Time course of (**a**) lateral tension σ and (**b**) the particle penetration ratio. The time 0 s indicates the time at which hypo-osmotic solution was added to the liposome solution.

We measured the time-dependent change in lateral tension and the particle penetration ratio for a single liposome (Figure 4). Membrane lateral tension was estimated by a change in the liposome surface area. Lateral tension increased just after the application of osmotic pressure at 0 to 250 s, and stayed between 0.02–0.03 N/m, as shown in Figure 4a. The temporary decrease in the tension at approximately 500 s may result from the formation of transient pores at the flat space of the membrane [15]. Conversely, the penetration ratio only began to increase 100 s after the increase in lateral tension, and became almost constant after 400 s (Figure 4b). We confirmed that all the liposomes with fully wrapped particles show the penetration when lateral tension of >0.01 N/m was applied (*N* = 11).

Next, we examined the dependence of penetration behavior on lateral tension. We applied several different osmotic pressures to induce different lateral tensions, and confirmed whether fully wrapped particles penetrated the membrane under five final osmotic pressures (Table 1). We determined six final lateral tensions (*σ*_f_) 600 s after the application of osmotic pressure, because these tensions became almost constant at 600 s, as shown in Figure 4a. Note that the negative penetration ratio of 0.003 N/m is attributed from error of measurements. We determined the penetration ratio by counting the number of fully wrapped particles on the membrane surface and those penetrated into the liposome from sectional images (the detailed calculation is indicated in the Materials and Methods section). Fluctuation of those particle numbers results in the error of measurements.

## 3. Discussion

**Figure 5:**
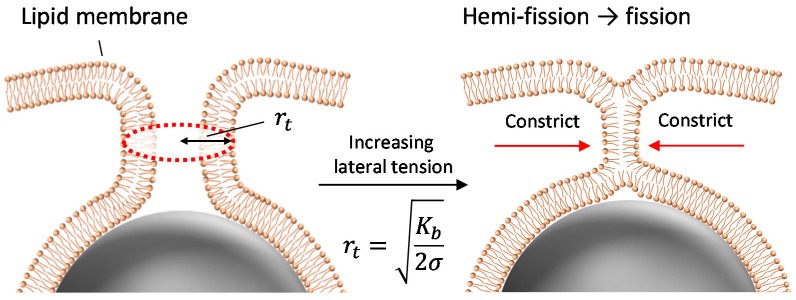
Schematic image of tube constriction and hemi-fission at the neck with increase in lateral tension.

The present results show that the application of lateral tension induces the penetration of fully wrapped particles into giant liposomes. The penetrated particles were covered by the membrane (as shown in Figure 2), which implies that the penetration was caused by membrane scission at the neck of a fully wrapped particle (Figure 5). Penetration was observed when applied lateral tension was over the order of 0.01 N/m (Table 1). Now we assume that the membrane neck consists of a tube structure with radius *r_t_,* as in Figure 5. In Reference [16], the tube radius *r_t_* is given by

(1) $$r_{t}=\sqrt{\frac{K_{b}}{2\sigma }}$$

where we assumed, for simplicity, that the neck tube is driven by the force of particle adhesion.

*K_b_* is bending modulus (~20 k_B_T, k_B_ is Boltzmann constant, T is room temperature) and σ is lateral tension. After the application of osmotic pressure, the tube radius decreased with increasing lateral tension. If we substitute σ ~ 0.01 N/m required to induce the particle penetration into Equation (1), *r_t_* ~ 2 nm, which is shorter than bilayer thickness (~4 nm). Thus, when the tube radius is constricted to become close to bilayer thickness, the tube probably transitions into a hemi-fission state. Further increase in lateral tension would generate the scission of hemi-fission structure so that wrapped particles penetrate into the liposome. The process from constricted tube to hemi-fission has been reported with dynamin-mediated membrane fission, where the driving force of constriction is the polymerization of dynamin [9]. Although here we found that fully wrapped particles show penetration when lateral tension of >0.01 N/m was applied, there is dispersion of the penetration ratio with the tension value (Figure 1). Two possible factors can be considered. (1) Fully wrapped particles prepared by applying centrifugal force have some differences in membrane deformation degree at the connection to a mother liposome, although there is no apparent difference in their microscopic images. The subtle difference in the connection may affect the tension-induced instability of the membrane neck; (2) Liposomes may initially have some tension, because sucrose concentration inside liposomes is stochastically determined during the process of liposome formation. It is difficult to determine absolute membrane tension including the initial tension, and we used relative tension value deduced from a change in liposomal size after the application of osmotic pressure. The initially tensed liposomes probably show high penetration ratio.

## 4. Materials and Methods 

DOPC (1,2-dioleoyl-sn-glycero-3-phosphocholine) was obtained from Avanti Polar Lipids (Alabaster, AL, USA). N-(rhodamine red-X)-1,2-dihexadecanoyl-sn-glycero-3-phosphoethanolamine triethylammonium salt (rho-DHPE) was obtained from Invitrogen (Waltham, MA, USA). Surfactant-free YG fluorescent polystyrene particles with a radius of 200 nm were obtained from Polyscience (Warrington, PA, USA). Giant unilamellar vesicles were made by the electro-formation method [17] using 100 mM sucrose solution. Liposomes with fully wrapped particles were obtained as previously reported [14]. First, we prepared 5.0 × 10^−3^ g/L polystyrene particles with YG-fluorescence in 100 mM glucose solution for the adhesion of particles to the liposome. To remove the potential for including residual surfactants in the particle solution, the solution was centrifuged (20,685 RCF, 30 min), and the supernatant was replaced with deionized water. This procedure was repeated three times. The particles were then dispersed in 100 mM glucose solution. Second, we mixed 30 µL liposome solution and 30 µL particle solution, where the particles and liposomes are heavier than the outer solution. Finally, we applied 1600 RCF centrifugal forces to the mixture for 10 min and obtained fully wrapped particles that were associated with liposomes (a video of fully wrapped particles is available in the SI). The number of the liposomes with fully wrapped particles obtained in this procedure was 3–10 in 3 μL.

**Figure 6:**
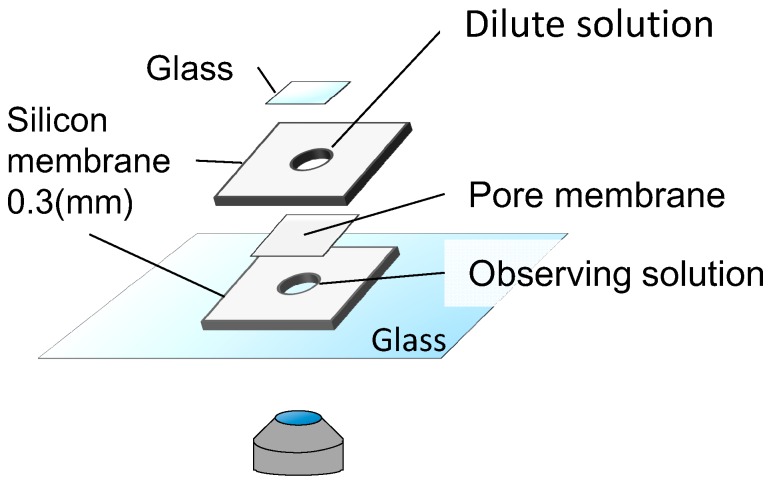
Schematic illustration of time-lapse observation under the application of tension.

To observe the dynamics of fully wrapped particles under lateral tension, we built a handmade chamber (Figure 6). First, we put a sample solution in the lower space of the chamber, and looked for membrane-associated particles within 5 min. Immediately after finding the liposomes, we focused on their equatorial plane to distinguish between fully wrapped and partially wrapped particles. Next, we filled the upper cylindrical space with 0 to 95 mM glucose solution to dilute the liposome solution, and placed a cover glass on the top. We then observed the liposome under the application of lateral tension for 20 min.

**Figure 7:**
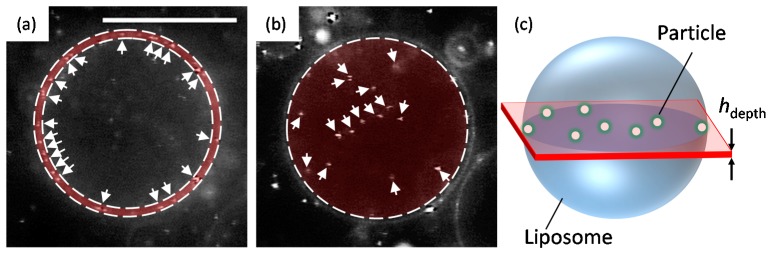
Regions for counting (**a**) fully wrapped particles and (**b**) penetrated particles. The white arrows indicate particles. Scale bar is 20 µm. (**c**) Schematic of the region observed by an optical microscope.

To estimate lateral tension, we measured the radius of liposomes. The lateral tension can be calculated as

(2) $$\sigma =K_{a}\left(\frac{\Delta S}{S_{0}}\right)$$

where $\Delta S=4\pi R^{2}-4\pi R_{0}^{2}$ is the difference between the liposome surface area under osmotic pressure and the initial surface area (*R*_0_ is the initial radius of a liposome and *R* is the radius of the liposome under osmotic pressure), $S_{0}=4\pi R_{0}^{2}$, and *K_a_* is the membrane elastic modulus of DOPC (0.265 ± 18 N/m) [18]. To determine the penetration ratio, we counted the number of fully wrapped particles on the membrane surface and the number of particles that had penetrated into the liposome from sectional images using Image-J version 1.49o (National Institutes of Health, Bethesda, MD, USA). Figure 7 shows the region for counting the average number of fully wrapped particles $N_{av}^{wrap}$ and the average number of penetrated particles $N_{av}^{pe}$ in 2000 consecutive frames (60 s: the value was estimated as *Δt* = (typical cross sectional area of liposomes)/(diffusion coefficient of 200 nm diameter particles)), respectively. The total number of particles in one liposome, $N^{wrap}$ and $N^{pe}$, are calculated as

(3) $$\frac{N_{AV}^{pe}}{\pi R^{2}h_{depth}}=\frac{N^{pe}}{\frac{4}{3}\pi R^{3}}$$

(4) $$\frac{N_{AV}^{wrap}}{2\pi Rh_{depth}}=\frac{N^{wrap}}{4\pi R^{2}}$$

The penetration ratio P is given by

(5) $$P=\frac{N^{pe}\left(t+\Delta t\right)-N^{pe}\left(0\right)}{N^{wrap}\left(0\right)}$$

where *t* indicates the duration of the application of osmotic pressure, $N^{pe}\left(t+\Delta t\right)$ is the number of penetrated particles at time *t* during *Δt* = 60 s, $N^{pe}\left(0\right)$ is the number of penetrated particles before the application of lateral tension, $N^{wrap}\left(0\right)$ is the number of fully wrapped particles before the application of lateral tension, and *h_depth_* is the depth of the focus (*h_depth_* ≈ 400 nm).

## 5. Conclusions

We prepared fully wrapped particles on a cell-sized liposome surface, and found that the particles entered the inner aqueous phase of the liposome under the application of lateral tension. The penetration was caused by tension-induced constriction of the neck part, which increases the probability of membrane hemi-fission. The scission of the hemi-fission leads to the detachment of fully wrapped particles. Our findings could lead to new technologies based on the mechanical properties of the membrane for transporting objects such as large plasmids and nanoparticles into a cell.

## Figures and Tables

**Table 1:** Relationship between the final lateral tension and penetration ratio.

Concentration Difference *ΔC* (C_in_ − C_out_) (mM)	2.5	10	10	12.5	25	50
Lateral tension *σ*_f_ (N/m)	0.003	0.017	0.027	0.032	0.027	0.026
Penetration ratio (%)	−0.6	4.7	100	6.2	7.9	69

## Supplementary Material

The following videos are available online at www.mdpi.com/1996-1944/10/7/765/s1, A video of fully wrapped particles on a membrane: Fully_wrapped_particles. A video of the process of penetration of fully wrapped particles under osmotic pressure: Penetration.
